# Intracranial manifestations of adult Rosai-Dorfman disease: a systematic review and IPD meta-analysis of 327 cases

**DOI:** 10.1007/s00701-025-06735-w

**Published:** 2025-12-06

**Authors:** Daniela A. Perez-Chadid, Aafreen Azmi, Jeremiah H. Wijaya, Temitope Oshinowo, Juan P. Avila-Madrigal, Aditi S. Gorthy, Sri Sai Lakshman Akkineni, Andrew Egladyous, Nemanja Novakovic, Morana Vojnic, Jonathan H. Sherman, Anil Nanda

**Affiliations:** 1 Department of Neurosurgery, New Jersey Medical School, Rutgers University, Newark, NJ USA; 2 Department of Neurosurgery, Robert Wood Johnson Medical School, Rutgers University, New Brunswick, NJ USA; 3 School of Public Health and Preventive Medicine, Monash University, Melbourne, VIC Australia; 4 School of Medicine and Health Sciences, Universidad del Rosario, Bogotá, Colombia; 5 Department of Medicine, Medical College of Georgia, Athens, GA USA; 6 Rutgers Cancer Institute, New Brunswick, NJ USA

**Keywords:** Intracranial Rosai–Dorfman disease, Central nervous system histiocytosis, Neurosurgical outcomes, Gross total resection, Neurosurgery, Neurology

## Abstract

Rosai–Dorfman disease (RDD) is a rare non-Langerhans histiocytosis that involves the central nervous system (CNS) in approximately 5% of cases, most often presenting intracranially. While pediatric cases have been reported more frequently, adult intracranial RDD (IC-RDD) remains poorly characterized. We conducted a systematic review and individual patient data meta-analysis in accordance with PRISMA guidelines to define the epidemiology, clinical presentation, pathology, management strategies, and outcomes of adult IC-RDD. PubMed, Scopus, and the Cochrane Library were searched for histologically confirmed cases in adults over 18 years, and case reports, case series, and observational studies were included. Data were extracted on demographics, symptoms, imaging, histopathology, treatment, and outcomes. Primary endpoints were recovery and recurrence-free survival (RFS), with risk ratios (RRs) and 95% confidence intervals (CIs) calculated using random-effects models. A total of 327 patients from 186 studies met inclusion criteria. The median age was 43.6 years (range 18–83), with a male predominance (70.9%). The most common symptoms were headache (35.2%), seizures or loss of consciousness (28.7%), and visual disturbance (26.9%). Lesions were typically supratentorial intra-axial (52.9%) or extra-axial (20.5%). Surgical intervention was performed in 93.6% of patients, with gross total resection (GTR) achieved in 45.3%. At a median follow-up of 18.8 months, 37.9% achieved full recovery, 46.8% partial recovery, and 15.3% experienced recurrence, with a median RFS of 12 months. GTR was strongly associated with improved recovery (RR 0.26, 95% CI 0.19–0.37), whereas supratentorial intra-axial location (RR 0.56, 95% CI 0.41–0.75) and perilesional edema (RR 0.65, 95% CI 0.47–0.89) predicted poorer outcomes. These findings indicate that adult IC-RDD predominantly affects middle-aged men, presents with mass-effect symptoms, and has a location-dependent prognosis, with GTR conferring the best chance of recovery but recurrence remaining common and underscoring the need for long-term surveillance.

**Supplementary Information:**

The online version contains supplementary material available at 10.1007/s00701-025-06735-w.

## Introduction

Rosai-Dorfman Disease (RDD) is a rare form of non-Langerhans cell histiocytosis characterized by the accumulation of distinctive histiocytes in affected tissues [2, 54]. RDD has an estimated prevalence of 1:200 000 and it is estimated that there are 100 new cases per year in the United States [2]. Extranodal disease occurs in more than 40% of cases; of these, around 5% represent central nervous system (CNS) manifestations. CNS RDD most frequently presents intracranially, with approximately 75% of CNS cases found in this location [2, 24].

Intracranial RDD often presents as a dural-based, avidly enhancing mass that is radiographically indistinguishable from several more common entities [24, 90]. The leading preoperative consideration is meningioma, but infectious, granulomatous, and autoimmune processes are also frequent differentials [2, 24, 54]. Clinical features are dictated by lesion location and mass effect, and can include headache, cranial neuropathies, seizures, or focal neurologic deficits. Because imaging is nonspecific, diagnosis rests on histopathology [54]. Characteristic findings include large pale histiocytes within a mixed inflammatory background that demonstrate S100 and CD68 positivity, lack CD1a expression, and display emperipolesis of intact lymphocytes and plasma cells [54].

Management of intracranial RDD is not standardized and is guided by case reports and small series. Surgical resection is the most common primary therapy for accessible or symptomatic lesions, often pursued to both obtain a diagnosis and relieve mass effect. Gross total resection has been associated with symptomatic improvement in many reports, although recurrence or progression can occur [15, 90]. When disease is multifocal, residual, or surgically high risk, clinicians have used corticosteroids, radiotherapy, and various systemic agents. Heterogeneity in patient selection, extent of resection, adjuvant therapy, and follow-up complicates the interpretation of outcomes [52].

Adult intracranial RDD remains insufficiently described because many reviews pool pediatric and adult patients or combine cranial and spinal disease, and pediatric disease differs enough to confound adult-specific conclusions. Important gaps persist regarding anatomic predilections, radiologic patterns that may prompt preoperative suspicion, determinants of surgical extent, and the effectiveness of adjuvant modalities. This systematic review focuses on adult intracranial RDD and aims to synthesize evidence on epidemiology, presentation, imaging, histopathology, treatment strategies, and clinical outcomes, including recurrence and complications. By consolidating fragmented literature, we seek to clarify current practice, identify areas of agreement and uncertainty, and outline priorities for prospective reporting and collaborative research.

## Methods

Our systematic review was conducted in accordance with the PRISMA (Preferred Reporting Items for Systematic Reviews and Meta-Analyses) guidelines [114]. We conducted a comprehensive literature search in PubMed, Scopus, and the Cochrane Library using a strategy that combined terms for “Rosai-Dorfman disease,” “sinus histiocytosis,” and neurosurgical keywords such as “intracranial,” “dura,” and “craniotomy,” as part of a broader three-part series into Rosai-Dorfman disease (RDD) of the central nervous system (CNS).

We included peer-reviewed case reports and case series that described adult patients (≥ 18 years) with histologically confirmed intracranial RDD. Individual cases from case series were only included when patient-level clinical, imaging, treatment, and outcome data were reported with sufficient detail to allow standardized extraction and reduce heterogeneity. Eligible studies had to report the location of intracranial lesions. We included only English-language publications and excluded editorials, reviews, and conference abstracts and studies lacking individual patient data. Studies without histopathologic confirmation were also excluded.

Two reviewers (DAPC and AA) independently screened titles and abstracts using Covidence. Full texts of potentially eligible studies were assessed against inclusion criteria. Discrepancies were resolved by discussion or, if needed, by a third reviewer (JPAM).

From each study, we extracted data on publication details, patient demographics, clinical presentation, imaging findings, and lesion characteristics. We also recorded surgical details, including approach and extent of resection. Pathologic features of interest included the presence of emperipolesis and immunohistochemical staining (S100, CD68, CD1a). We noted any adjuvant therapies (e.g., corticosteroids, radiation, chemotherapy) and summarized outcomes such as neurologic recovery, recurrence, follow-up duration, and mortality.

We assessed study quality using the Joanna Briggs Institute (JBI) critical appraisal tools for case reports and case series. While no studies were excluded based on quality, these assessments informed our interpretation of the findings. A summary of scores and risk-of-bias domains is provided in Supplement 1 to contextualize interpretation of pooled findings. Ethics or IRB approval was not required due to the review nature of our paper. All included patient information was obtained from already published case reports and case series. Due to this individual patient consent was not possible as data was already published as de-identified cases.

### Statistical analysis

All analyses were conducted in R (v4.5.1) within RStudio (stable release 2025.05 “Mariposa Orchid”) on macOS. Categorical variables were summarized as n (%) and continuous variables as median (min–max). The age–sex distribution was visualized as a population pyramid built with ggplot2 (within tidyverse) after binning age into predefined intervals; counts for males were plotted as negative values to mirror females (packages: tidyverse/ggplot2). Recurrence-free survival (RFS) was estimated with Kaplan–Meier methods using survival and survminer (median RFS with 95% CIs; log-rank and Cox models applied for subgroup comparisons). For pooled effects on binary outcomes, we calculated risk ratios (RRs) using random-effects models (REML) via metafor (cross-checked with meta for sensitivity); heterogeneity was quantified with τ^2^ and I^2^, and study effects were explored with funnel plots and Egger’s regression. The heatmap of IHC marker frequencies by lesion location was produced with pheatmap (Euclidean distance, complete linkage; red palette scaled to counts).

Study-specific best linear unbiased predictions (BLUPs) were derived from the fitted mixed-effects model to estimate study-level deviations from the pooled effect. A caterpillar plot was generated to visualize the BLUPs, showing each study’s predicted effect and corresponding 95% confidence interval, providing a comparative view of study-level deviations within the overall model. Figures were finalized in R using ggplot2.

## Results

**Fig. 1:**
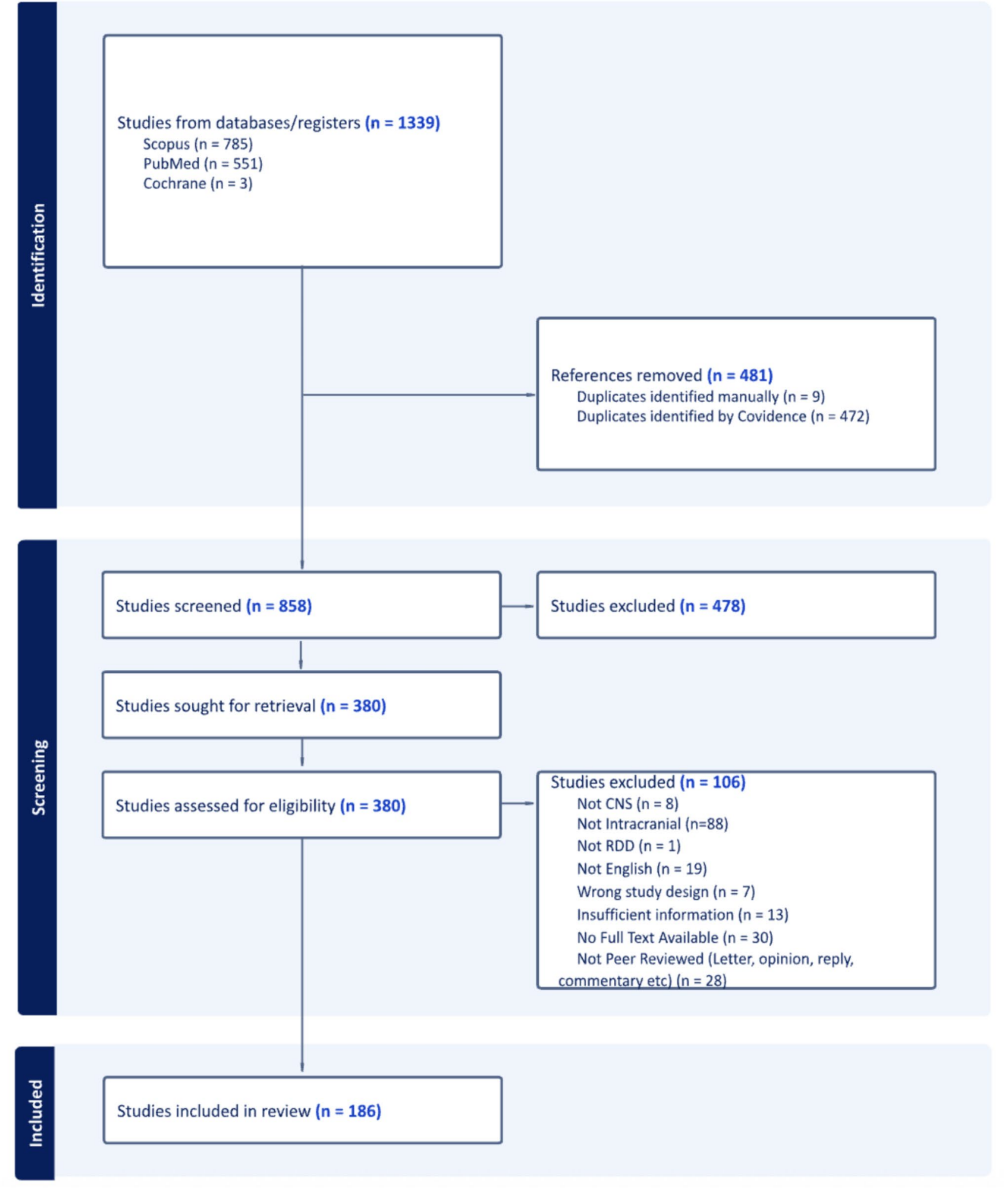
PRISMA diagram

After duplicate removal, a total of 858 studies were identified for screening. Upon conducting a thorough review of titles, abstracts, and full texts, 186 studies were selected for inclusion in our final analysis (Fig. 1).

**Fig. 2:**
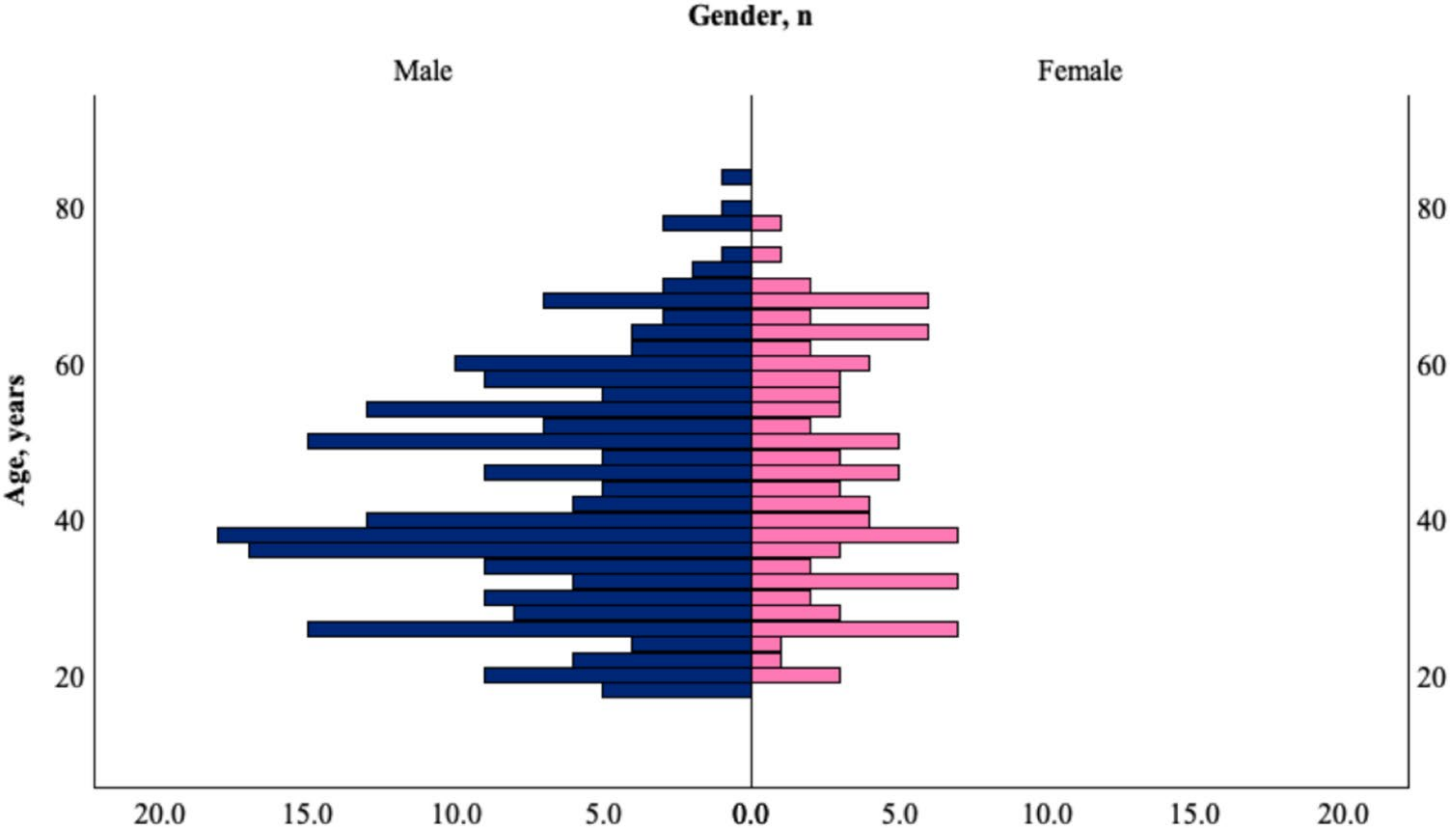
Age and sex distribution of the study population

**Fig. 3:**
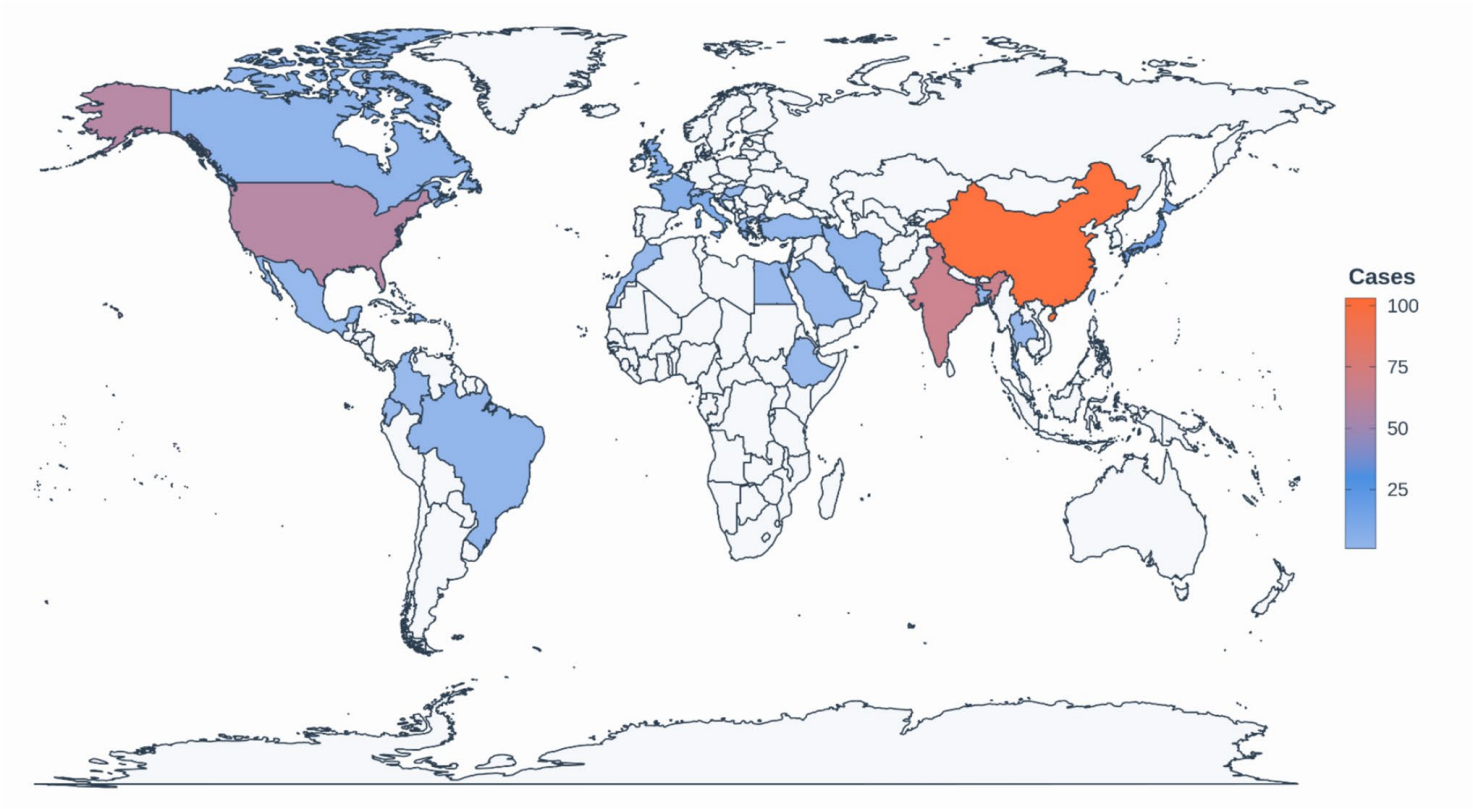
Geographical distribution of included adult IC-RDD cases

In this review, a total of 327 patients were included, with a predominance of males (232, 70.9%) and a median age of 43.6 years (range 18–83). The age- and sex-distribution showed that cases were concentrated in middle adulthood, with a male predominance across nearly all age groups, particularly in the 30–50 year range (Fig. 2). The country with the most reported cases was China with 103 (31.%) reported cases, followed by India with 68 cases (20.8%) and USA with 58 (17.7%) cases (Fig. 3).

The most common presenting symptom was headache (115, 35.2%), followed by seizure or loss of consciousness (94, 28.7%) and visual disturbance (88, 26.9%), whereas cranial nerve palsy (17.1%) and cognitive/language changes (7.0%) were less frequently reported. Tumors were most often located in the supratentorial region (intra-axial: 52.9%, extra-axial: 20.5%), with sellar/parasellar (20.8%) and infratentorial (18.0%) lesions also frequent. MRI was the predominant imaging modality (85%), while PET and angiography were rarely used. Perilesional edema was observed in 12.2% of patients, and hydrocephalus was uncommon (2.1%). Preoperative diagnostic impressions were documented for 225 of 327 intracranial RDD cases (68.8%). In 166 cases (50.8% of the full cohort, 73.8% of cases with a stated differential), meningioma was specifically listed as part of the differential diagnosis. Almost all patients underwent surgery (93.6%), most commonly via craniotomy (76.1%), with gross total resection achieved in 45.3%. Spinal involvement occurred in 10.7% of cases, most frequently in the cervical spine. Postoperative complications were reported in 9.8% of patients, and re-operation was required in 7.0%.

Histopathological confirmation was consistent, with emperipolesis (57.5%), inflammatory infiltrate (77.1%), and histiocytes (74.6%) being the most prevalent features. Immunohistochemistry showed high rates of S100 (87.5%) and CD68 (64.5%) positivity, while CD1a was positive in nearly half (47.4%), reflecting the heterogeneity of marker expression. Adjuvant therapies included corticosteroids (25.1%), radiotherapy (13.5%), and chemotherapy (6.7%), while a subset (10.7%) received no adjuvant treatment. At follow-up (median 18.8 months, range 0–120), full recovery was observed in 37.9% of patients, partial recovery in 46.8%, and stable disease in 25.4%, while recurrence occurred in 15.3% with a median time to recurrence of 2.4 months. All-cause mortality remained relatively low at 4.9%; death attributed to surgical complications was 3.7%. Table 1 presents the demographic characteristics of the study population.

**Table 1:** Demographic and clinical characteristics of included patients (*n* = 327)

Category		Variable	*n* (%)
Sex		Male	232 (70.9)
Age, years, median (min–max)			43.55 [18–83]
Clinical presentation			
	Presenting symptoms	Headache	115 (35.2)
		Seizure/LOC	94 (28.7)
		Visual disturbance	88 (26.9)
		Motor/Sensory deficit	71 (21.7)
		Cranial nerve palsy	56 (17.1)
		Cognitive/Language change	23 (7.0)
		Constitutional/Systemic	33 (10.1)
		Nausea/Vomiting/Dizziness	39 (11.9)
		Endocrine	3 (0.9)
	Tumor location	Supratentorial—Intra-axial	173 (52.9)
		Supratentorial—Extra-axial	67 (20.5)
		Sellar/Parasellar	68 (20.8)
		Infratentorial	59 (18.0)
		Ventricular	12 (3.7)
		Skull base/Sinonasal/Orbit	42 (12.8)
		Other/Unclassified	18 (5.5)
	Imaging used	MRI	278 (85.0)
		CT	109 (33.3)
		PET	14 (4.3)
		Angiography	1 (0.3)
		Other imaging	2 (0.6)
	Peri-tumoral	Perilesional edema	40 (12.2)
	Hydrocephalus/EVD	Hydrocephalus	7 (2.1)
		EVD/VPS placed	3 (0.9)\†
Initial management		Surgery (any)	306 (93.6)
		Observation	5 (1.5)
	Surgical approach (subtypes)	Craniotomy	249 (76.1)
		SRS/biopsy	4 (1.2)
		Biopsy only	21 (6.4)
		Endoscopic sinus	4 (1.2)
		Other surgery	14 (4.3)
	Resection category	Gross total resection	148 (45.3)
		Subtotal resection (STR)	60 (18.3)
		Partial resection	28 (8.6)
		Biopsy only	29 (8.9)
	Spine involvement	Any spinal involvement	35 (10.7)
		Cervical	20 (6.1)
		Thoracic	12 (3.7)
		Lumbar	8 (2.4)
	Outcomes/Events	Complications	32 (9.8)
Post-op imaging result		Complete response	61 (18.7)
		Stable	22 (6.7)
		Improved	36 (11.0)
		Infarcts	1 (0.3)
Disease pattern		Multisystem RDD	51 (15.6)
Extranodal sites		Lymph nodes	20 (6.1)
		Orbit	19 (5.8)
		Paranasal sinuses/nasal	23 (7.0)
		Skin/soft tissue	6 (1.8)
		Thoracic	14 (4.3)
		Abdominal/GI/solid organ	15 (4.6)
		Other extranodal	3 (0.9)
Post-operative outcomes			
	Surgery	Re-operation rate	23 (7.0)
	Adjuvant therapy	Corticosteroid	82 (25.1)
		Radiotherapy	44 (13.5)
		Chemotherapy	22 (6.7)
		Antiepileptic drugs (AED)	13 (4.0)
		No adjuvant therapy	35 (10.7)
		IVIG	3 (0.9)
		6-mercaptopurine	4 (1.2)
		Other adjuvant	6 (1.8)
Pathology			
	IHC markers (positive)	S100	286 (87.5)
		CD68	211 (64.5)
		CD163	11 (3.4)
		GFAP	8 (2.4)
		CD138	3 (0.9)
		L26	1 (0.3)
		KP1	1 (0.3)
		EMA	8 (2.4)
		CD31	3 (0.9)
		CD3	2 (0.6)
		Vimentin	6 (1.8)
		Fibronectin	1 (0.3)
		Anti-SSA	1 (0.3)
		Anti-SSB	1 (0.3)
		ANA	1 (0.3)
		Langerin	7 (2.1)
		Ki-67	2 (0.6)
		CD1a	155 (47.4)
	Histopathology (positive)	Emperipolesis	188 (57.5)
		Inflammatory infiltrate	252 (77.1)
		Fibrosis	60 (18.3)
		Histiocytes	244 (74.6)
		MALT features	1 (0.3)
		Necrosis	4 (1.2)
		Eosinophil granules	5 (1.5)
		Lymphocytic proliferation	3 (0.9)
		Giant cells	3 (0.9)
		Other histo features	1 (0.3)
	Mutations	BRAF V600E	3 (0.9)
		KRAS	1 (0.3)
Outcomes		Full recovery	124 (37.9)
		Stable disease	83 (25.4)
		Death	16 (4.9)
		Systemic disease	1 (0.3)
		Progressive disease	18 (5.5)
		Partial recovery	153 (46.8)
		Recurrence	50 (15.3)
Time to recurrence, months, median (min–max)			2.39 (0–84)
Follow-up, months, median (min–max)			18.81 (0–120)

Follow-up was available for 216 patients (66.1%), with a median of 18.0 months (IQR 8.0 to 43.2). Clinical status at last follow-up was reported for 271 patients: full recovery 124 (45.8%), stable disease 83 (30.6%), partial recovery 29 (10.7%), progressive disease 18 (6.6%), and death 16 (5.9%). Recurrence status was available for 218 patients, with recurrence in 50 (22.9%). Time to recurrence was reported for 46 patients, with a median of 11.5 months (IQR 3.8 to 20.8). Death attributed to RDD was recorded in 11 patients.

**Fig. 4:**
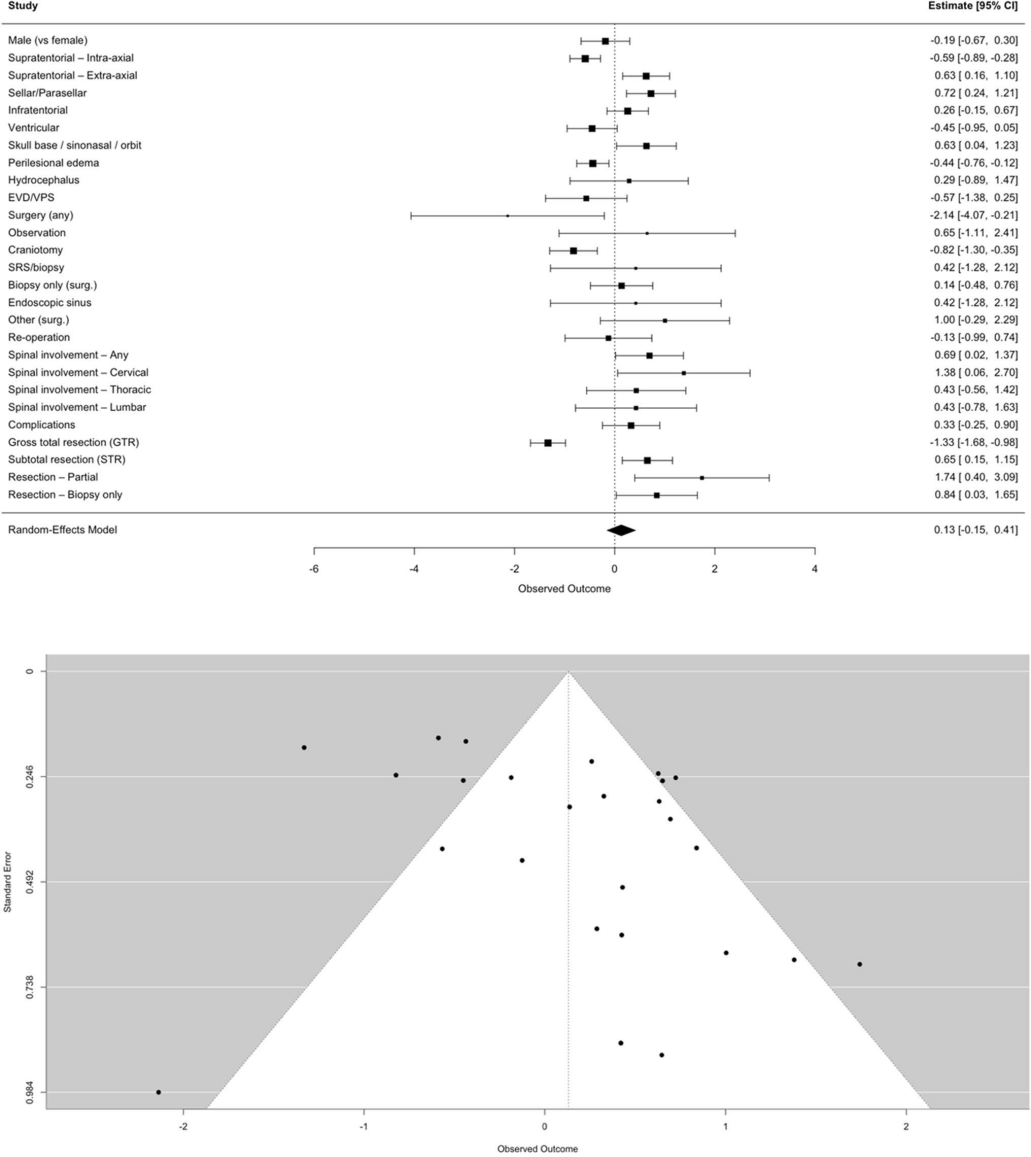
Forest and funnel plot for the individual patient level data analysis

In the individual patient data meta-analysis (Table 2 and Fig. 4), male sex was not associated with full recovery (RR 0.83, 95% CI 0.51–1.35, *p* = 0.455). Lesion location emerged as an important predictor: supratentorial intra-axial lesions were significantly less likely to achieve full recovery (RR 0.56, 95% CI 0.41–0.75, *p* < 0.001), whereas supratentorial extra-axial (RR 1.87, 95% CI 1.17–2.99, *p* = 0.003), sellar/parasellar (RR 2.06, 95% CI 1.27–3.36, *p* < 0.001), and skull base/sinonasal/orbit lesions (RR 1.88, 95% CI 1.04–3.42, *p* = 0.018) were significantly associated with higher rates of recovery. In contrast, infratentorial and ventricular locations did not reach statistical significance. Among perioperative factors, perilesional edema was linked to reduced recovery (RR 0.65, 95% CI 0.47–0.89, *p* = 0.017), while hydrocephalus was not predictive (RR 1.33, 95% CI 0.41–4.34, *p* = 0.606). Prior surgery was significantly associated with worse outcomes (RR 0.12, 95% CI 0.02–0.81, *p* = 0.001), whereas EVD/VPS placement and observation showed no significant effect. Regarding surgical management, craniotomy was associated with improved recovery (RR 0.44, 95% CI 0.27–0.71, *p* < 0.001), while other approaches, including SRS/biopsy, endoscopic sinus, and biopsy alone, were not significant. Spinal involvement was overall predictive of poorer outcomes (RR 2.00, 95% CI 1.02–3.94, *p* = 0.021), particularly in cervical lesions (RR 3.97, 95% CI 1.06–14.91, *p* = 0.008). Finally, extent of resection played a critical role: gross total resection was strongly associated with improved recovery (RR 0.26, 95% CI 0.19–0.37, *p* < 0.001), while subtotal resection (RR 1.92, 95% CI 1.16–3.17, *p* = 0.004), partial resection (RR 5.71, 95% CI 1.49–21.87, *p* < 0.001), and biopsy only (RR 2.32, 95% CI 1.03–5.20, *p* = 0.016) were all associated with worse outcomes.

**Table 2:** Individual patient data meta-analysis of predictors for full recovery

Variable	RR	95% CI	p-value
Male	0.83*	0.51–1.35	0.455
Lesion location			
Supratentorial – Intra-axial	0.555*	0.409–0.753	< 0.001
Supratentorial – Extra-axial	1.873	1.172–2.991	0.003
Sellar/Parasellar	2.063	1.267–3.360	< 0.001
Infratentorial	1.296	0.858–1.959	0.196
Ventricular	0.637*	0.386–1.049	0.138
Skull base/sinonasal/orbit	1.883	1.038–3.418	0.018
Perilesional edema	0.646*	0.469–0.891	0.017
Hydrocephalus	1.334	0.410–4.341	0.606
Previous management			
EVD/VPS	0.567*	0.251–1.277	0.306
Surgery	0.118*	0.017–0.806	0.001
Observation	1.910	0.329–11.085	0.405
Surgical Management			
Craniotomy	0.439*	0.273–0.707	< 0.001
SRS/biopsy	1.523	0.277–8.364	0.592
Biopsy only	1.147	0.616–2.135	0.655
Endoscopic sinus	1.523	0.277–8.364	0.592
Other	2.728	0.751–9.917	0.062
Re-operation	0.882*	0.371–2.099	0.786
Spinal involvement			
Any	2.003	1.018–3.944	0.021
Cervical	3.974	1.059–14.906	0.008
Thoracic	1.537	0.571–4.135	0.347
Lumbar	1.530	0.457–5.121	0.446
Complications	1.386	0.782–2.456	0.229
Resection			
Gross total resection (GTR)	0.264*	0.186–0.374	< 0.001
Subtotal resection (STR)	1.919	1.162–3.169	0.004
Partial	5.712	1.492–21.869	< 0.001
Biopsy only	2.316	1.031–5.204	0.016

An asterisk (*) indicates variables with risk ratios (RR) < 1, which suggest an increased likelihood of full recovery

**Fig. 5:**
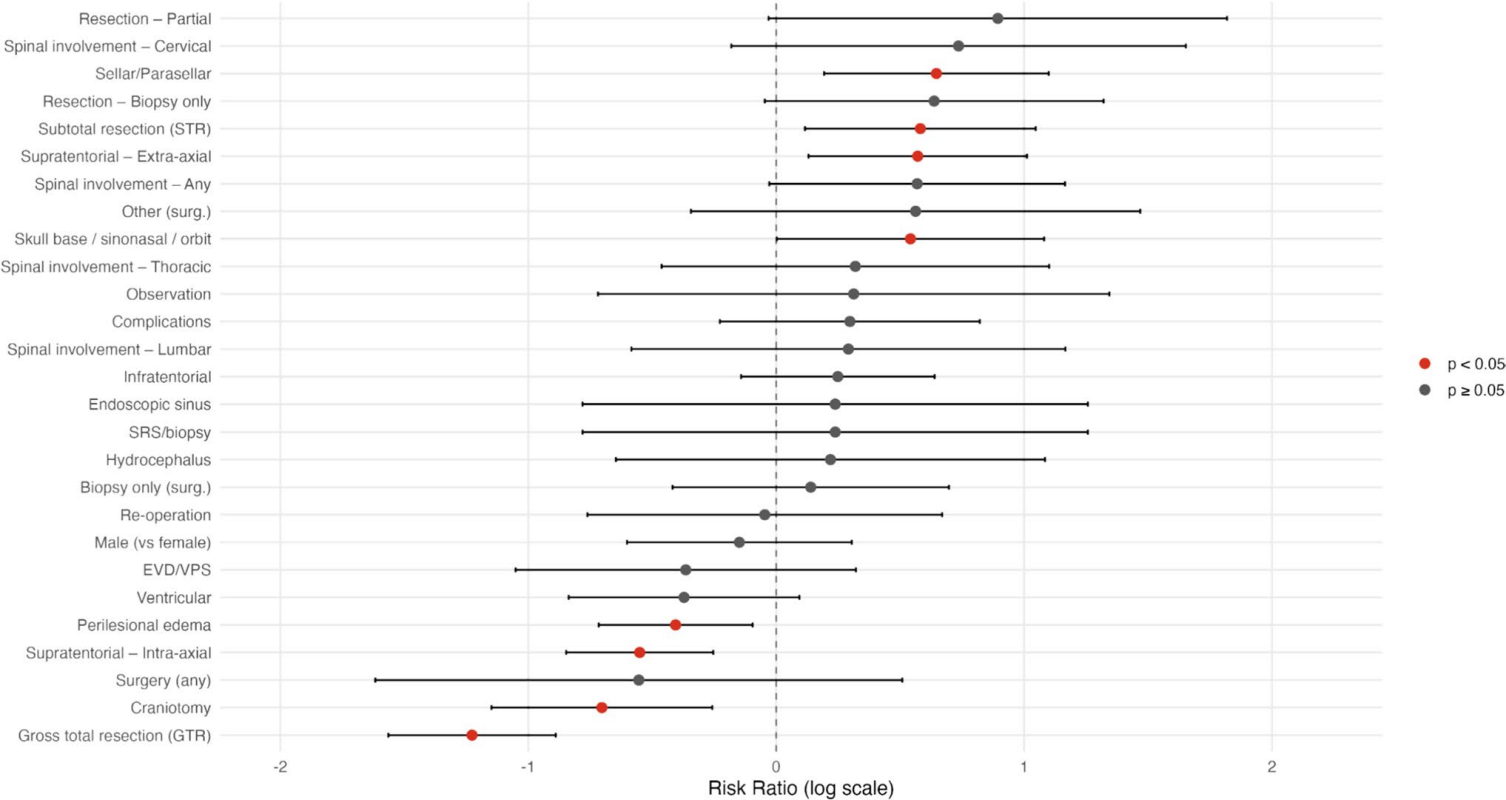
Caterpillar plot of BLUPs, summarizing study-specific random effects from the one-stage mixed-effects analysis

We estimated effects using BLUP (Table 3) from a random-effects meta-analysis of RR. Each BLUP represents the model-predicted true effect for a study, combining its observed log-RR and precision with the pooled mean (μ̂ = 0.14) while accounting for between-study heterogeneity (τ^2^ = 0.422). Imprecise studies are more strongly shrunk toward the overall mean, whereas precise studies remain close to their observed effects. Points to the right of 0 indicate increased risk (RR > 1), and points to the left of 0 decreased risk (RR < 1); red markers (*p* < 0.05) indicate statistically significant deviations from no effect (Fig. 5).

**Table 3:** Included Cases

Author	Age (years), Sex	Presenting Symptoms	Location	Differential Diagnoses	Adjuvant Therapy	Clinical Outcome
Abdel-Razek 2013 [1]	43 M	Headache, Seizure, Visual disturbance, Dizziness	Frontal lobe	Meningioma		
Abdel-Razek 2013 [1]	38 F	Seizure, Papilledema	Parasagittal, Parietal lobe	Meningioma		
Algül 2024 [4]	32 M	Visual disturbance, Headache	Cerebellum, Pons, Cerebello-pontine angle		Antiepileptic	Stable disease
Aljohani 2018 [5]	67 M	Headache, Visual disturbance	Suprasellar	Meningioma		
Almási 2023 [6]	53 M	Other, Seizure	Meninges	Meningioma, Other		
Ambekar 2011 [7]	37 F	Visual disturbance	Medulla	Meningioma, Lymphoma, Tuberculosis, Other	Radiation therapy	
Andriko 2001 [8]	50 M	Headache	Skull base, Dura	Hamartoma		Stable disease
Andriko 2001 [8]	22 M	Seizure	Frontal lobe, Temporal lobe, Parietal lobe	Meningioma		Stable disease
Andriko 2001 [8]	63 F	Headache	Frontal lobe			
Andriko 2001 [8]	25 F	Headache	Falx cerebri			Stable disease
Andriko 2001 [8]	31 F	Seizure, Numbness	Falx cerebri			Death
Andriko 2001 [8]	62 M	Visual disturbance	Sellar, Skull base	Lymphoma		Partial recovery
Andriko 2001 [8]	43 F	Headache	Parietal lobe	Inflammation		Full recovery
Andriko 2001 [8]	24 M	Seizure, Loss of Consciousness	Occipital lobe, Dura	Meningioma		Full recovery
Aradhana 2018 [9]	35 M	Headache, Visual disturbance	Other	Meningioma	Corticosteroids, Radiation therapy, Chemotherapy (nonspecific)	Death
Aradhana 2018 [9]	57 M	Aphasia	Parietal lobe, Frontal lobe	Meningioma	Radiation therapy	Full recovery
Aradhana 2018 [9]	37 M	Weakness	Brainstem	Other	Corticosteroids, Radiation therapy	Partial recovery
Arnao 2016 [11]	70 M	Seizure, Visual disturbance, Other, Dizziness, Movement disorder	Frontal lobe, Parietal lobe, Temporal lobe, Sellar, Other	Meningioma	Corticosteroids, Chemotherapy (nonspecific)	Stable disease
ArunKumar 2014 [12]	43 M	Loss of Consciousness	Temporal lobe, Frontal lobe	Meningioma, Glioma		
Asai 1988 [13]	39 M	Visual disturbance	Occipital lobe			Stable disease
Asiri 2023 [14]	37 F	Seizure	Parietal lobe, Extra axial	Meningioma	Antiepileptic	Full recovery
Balcha 2023 [16]	19 F	Neck Swelling, Painless mass	Middle fossa, Posterior fossa, Parasellar, Cerebellopontine angle	Lymphoma, Metastasis, Meningioma		
Benson 2025 [17]	63 F	Ataxia/gait disturbance, Visual disturbance, Other	Suprasellar	Lymphoma, Metastasis, Other		Partial recovery
Beros 2011 [18]	41 M	Dizziness/Vertigo	Cerebellum	Glioma, Sarcoidosis, Metastasis		Full recovery
Bezerra Lima 2019 [19]	60 F	Seizure	Parietal lobe		Steroid therapy	Full recovery
Bhat 2015 [20]	38 M	Headache	Parietal lobe	Meningioma, Glioma, Tuberculosis, Syphilis, Metastasis, Sarcoidosis		
Bhattacharjee 1992 [21]	78 M	Visual disturbance, Cranial nerve palsy	Frontal lobe, Suprasellar			Full recovery
Bing 2009 [22]	32 F	Seizure, Visual disturbance, Movement disorder	Occipital lobe, Parietal lobe, Ventricular		Corticosteroids	Full recovery
Boissaud-Cooke 2020 [23]	52 M	Nausea/vomiting, Dizziness/Vertigo	Frontal lobe			
Bueno 2022 [25]	50 M	Visual disturbance	Frontal lobe, Dura	Meningioma		
Bueno 2022 [25]	35 M	Visual disturbance	Skull base		Corticosteroids, Radiation therapy	Partial recovery
Burgos-Sosa 2024 [26]	57 M	Headache	Frontal lobe, Orbit, Paranasal Sinus			
Camp 2012 [27]	31 F	Seizure	Frontal lobe			Full recovery
Cao 2011 [28]	35 M	Visual disturbance, Weakness	Ventricular	Meningioma		Full recovery
Cao 2023 [29]	59 M	Movement disorder	Medulla			Partial recovery
Cao 2023 [29]	35 M	Cranial nerve palsy, Visual disturbance	Medulla			Death
Carey 1987 [30]	35 M	Painless mass, Weight Loss	Parasagittal	Meningioma		Stable disease
Catalucci 2012 [31]	57 M	Visual disturbance, Tinnitus	Ventricular, Extra axial	Meningioma		Systemic Disease
Cavuoto 2011 [32]	25 M	Headache, Painless mass, Dizziness/Vertigo, Visual disturbance, Seizure	Pons, Cerebellum, Temporal lobe, Frontal lobe, Basal Nuclei, External Capsule	Disseminated Encephalomyelitis	Intravenous Immunoglobulin, Steroid therapy	Stable disease
Chang 2003 [33]	46 M	Nasal Obstruction, Epistaxis	Nasal Cavity, Paranasal Sinus, Cavernous Sinus, Dura	Granuloma		Stable disease
Chen 2003 [33]	70 M	Headache	Parietal lobe, Suprasellar			Death
Chen 2014 [12]	41 F	Tender lump	Extra axial, Skull	Meningioma, Metastasis	Steroid therapy	
Cheng 2018 [34]	41 F	Headache, Dizziness/Vertigo, Nausea/vomiting	Temporal lobe	Glioma		Full recovery
Cheng 2018 [34]	63 F	Cognitive changes, Nausea/vomiting	Cerebellum	Intracerebral hemorrhage		Full recovery
Cheng 2018 [34]	35 F	Nausea/vomiting, Cognitive changes	Frontal lobe	Meningioma		Full recovery
Cheng 2018 [34]	20 F	Headache, Dizziness/Vertigo	Temporal lobe	Meningioma		Full recovery
Cheng 2018 [34]	37 M	Headache, Cognitive changes	Temporal lobe	Lymphoma		Full recovery
Cheng 2018 [34]	60 M	Headache, Nausea/vomiting	Cerebellum	Meningioma		Full recovery
Cheng 2018 [34]	59 M	Headache, Weakness	Cerebellum	Lymphoma		Full recovery
Chivukula 2015 [35]	66 F	Headache, Visual disturbance, Cognitive changes, Nerve Palsy	Hypothalamus			
Chong 2021 [36]	61 M	Headache	Sellar, Suprasellar, Skull base, Posterior fossa	Meningioma		Full recovery
Chougule 2024 [37]	20 M	Cognitive changes	Intraventricular, Fourth ventricle	Ependymoma, Medulloblastoma, Glioneural Tumor		Full recovery
D'Oria 2024 [39]	51 M	Ataxia/gait disturbance, Spasticity, Aphasia	Occipital lobe, Pons, Temporal lobe, Frontal lobe, Parietal lobe		Steroid therapy, Radiation therapy	Partial recovery
Das 2017 [40]	50 M	Visual disturbance, Loss of Consciousness	Pituitary stalk, Suprasellar, Extra axial	Meningioma	Steroid therapy, Radiation therapy	Progressive disease
Deodhare 1998 [42]	41 M	Seizure	Parietal lobe, Occipital lobe	Meningioma		Full recovery
Deodhare 1998 [42]	38 M	Headache, Seizure, Loss of Consciousness	Parietal lobe, Occipital lobe	Meningioma		Full recovery
Dodson 2003 [43]	56 F	Headache, Seizure	Parietal lobe, Nasal Cavity			Full recovery
Elshikh 2020 [44]	63 M		Other	Meningioma		
Elshikh 2020 [44]	19 M		Skull base, Suprasellar, Sellar	Meningioma		
Elshikh 2020 [44]	54 M		Pons, Medulla, Suprasellar, Sellar	Meningioma		
Feng 2022 [46]	65 M	Cognitive changes	Optic Nerves/Chiasm	Glioma		Death
Feng 2022 [46]	61 F	Dizziness/Vertigo, Cognitive changes, Headache	Parietal lobe	Meningioma		
Forest 2014 [47]	41 M	Seizure	Temporal lobe, Frontal lobe	Meningioma, Inflammation	Antiepileptic	Stable disease
Forest 2014 [47]	35 F	Visual disturbance, Cranial nerve palsy	Optic Nerves/Chiasm, Orbit	Meningioma		Stable disease
Forest 2014 [47]	38 M	Cognitive changes, Seizure	Falx cerebri	Meningioma, Glioneural Tumor		Stable disease
Franco-Paredes 2002 [48]	57 F	Seizure, Movement disorder	Meninges	Meningioma, Other	Corticosteroids	Full recovery
Friconnet 2021 [49]	30 M	Weight Loss, Nausea/vomiting, Dizziness/Vertigo, Cranial nerve palsy	Cerebellum, Fourth ventricle	Meningioma	MEK inhibitors, BRAF inhibitors	Full recovery
Fukushima 2011 [50]	33 F	Headache	Frontal lobe, Parietal lobe	Lymphoma, Glioma, Metastasis	Corticosteroids	Full recovery
Gaetani 2000 [51]	67 F	Ataxia/gait disturbance	Cerebellum, Fourth ventricle	Meningioma		Full recovery
Ghosal 2012 [53]	36 M	Seizure, Visual disturbance	Frontal lobe, Parietal lobe	Meningioma		Full recovery
Ghosal 2012 [53]	26 M	Seizure, Visual disturbance	Parietal lobe	Meningioma		Full recovery
Griauzde 2013 [55]	26 M	Nausea/vomiting, Visual disturbance	Temporal lobe	Langerhans cell histiocytosis, Meningioma, Metastasis		
Guarnizo 2022 [56]	19 M	Visual disturbance, Dizziness/Vertigo	Dura, Pons, Suprasellar, Cerebello-pontine angle	Meningioma		
Hadjipanayis 2003 [57]	52 M	Headache, Fever, Visual disturbance, Cranial nerve palsy	Cavernous Sinus, Posterior fossa	Meningioma	Corticosteroids	Stable disease
Hashimoto 2014 [58]	53 M	Painless mass	Frontal lobe, Dura, Temporal lobe	Lymphoma	Corticosteroids	Full recovery
Hinduja 2009 [61]	42 M	Seizure, Visual disturbance, Cranial nerve palsy	Optic Nerves/Chiasm, Cavernous Sinus, Middle fossa	Sarcoidosis, Meningioma, Erdheim-Chester disease	Corticosteroids, Radiation therapy	Partial recovery
Hingwala 2011 [62]	36 M	Visual disturbance, Headache, Cranial nerve palsy	Posterior fossa			
Hollon 2016 [63]	26 M	Dizziness/Vertigo, Nausea/vomiting, Visual disturbance	Temporal lobe	Meningioma, Granuloma, Hemangiopericytoma, Other		Full recovery
Hong 2016 [64]	59 F	Seizure	Cerebellum	Ependymoma, Glioneural Tumor, Metastasis, Lymphoma		Full recovery
Hong Cheng 2017 [65]	64 M	Seizure	Frontal lobe	Meningioma		
Huang 1998 [66]	38 M	Loss of Consciousness, Seizure	Parietal lobe	Meningioma		Full recovery
Huang 2016 [68]	18 M	Seizure	Parietal lobe, Occipital lobe, Optic Nerves/Chiasm, Hypothalamus	Meningioma		Partial recovery
Huang 2016 [68]	18 M	Seizure, Numbness	Skull base, Posterior fossa	Meningioma		Partial recovery
Huang 2016 [68]	50 F	Seizure	Parietal lobe	Meningioma		Full recovery
Huang 2016 [68]	40 M	Seizure	Parietal lobe	Meningioma		Full recovery
Huang 2016 [68]	68 M	Tender lump	Frontal lobe	Granuloma		Full recovery
Huang 2024 [67]	54 M	Movement disorder	Basal ganglia, Thalamus		Corticosteroids	Death
Imada 2015 [69]	68 F	Visual disturbance	Lateral ventricles, Hypothalamus	Sarcoidosis, Lymphoma, Tuberculosis, Metastasis	Corticosteroids	Progressive disease
Jiang 2018 [70]	39 M	Seizure, Headache	Frontal lobe, Parietal lobe	Meningioma	Radiation therapy	Stable disease
Jiang 2018 [70]	53 M	Seizure	Parietal lobe, Occipital lobe, Temporal lobe	Meningioma		Stable disease
Jin 2021 [71]	33 M	Seizure	Frontal lobe, Temporal lobe, Parietal lobe	Meningioma	Radiation therapy	Full recovery
Joshi 2019 [72]	42 F	Seizure	Parietal lobe	Meningioma		Stable disease
Joshi 2019 [72]	40 M	Headache, Seizure	Parietal lobe			Full recovery
Joshi 2019 [72]	46 M	Seizure	Parietal lobe			Full recovery
Joshi 2019 [72]	58 M	Headache, Nerve Palsy, Cognitive changes, Weakness	Occipital lobe, Temporal lobe			Partial recovery
Juan 2023 [73]	44 F	Visual disturbance	Suprasellar	Lymphoma, Metastasis, Other, Langerhans cell histiocytosis		Partial recovery
Jurić 2003 [74]	39 M	Dizziness/Vertigo, Visual disturbance	Temporal lobe	Meningioma		Full recovery
Kattner 2000 [77]	33 M	Headache, Seizure	Parasagittal, Frontal lobe, Parietal lobe	Meningioma		Full recovery
Kapoor 2018 [76]	58 M	Weight Loss, Neck Swelling	Fourth ventricle, Pons, Medulla		6-mercaptopurine, Corticosteroids	Full recovery
Kattner 2000 [77]	33 M	Headache	Parasagittal	Meningioma	Corticosteroids	Full recovery
Katz 1993 [78]	20 M	Weight Loss, Nausea/vomiting	Posterior fossa			Stable disease
Kelly 1999 [79]	45 F	Headache, Fever, Visual disturbance	Pituitary gland, Suprasellar, Optic Nerves/Chiasm	Other	Corticosteroids, Radiation therapy	Stable disease
Kidd 2006 [80]	37 F	Headache, Visual disturbance	Parasellar	Meningioma, Sarcoidosis	Radiation therapy	Stable disease
Kidd 2006 [80]	68 M	Visual disturbance	Suprasellar, Parasellar		Radiation therapy	Partial recovery
Kim 1995 [81]	50 M	Seizure, Movement disorder, Visual disturbance	Parietal lobe, Occipital lobe	Meningioma	Corticosteroids, Antiepileptic	Full recovery
Kitai 1996 [83]	25 M	Headache, Seizure	Occipital lobe	Meningioma	Corticosteroids, Radiation therapy	Full recovery
Kitai 2001 [82]	25 M	Headache, Dizziness/Vertigo	Occipital lobe		Corticosteroids, Other	Full recovery
Kitai 2001 [82]	36 M	Headache, Seizure	Frontal lobe			
Kitai 2001 [82]	42 F	Headache, Seizure	Tenorital			
Konishi 2003 [84]	68 F	Seizure, Cranial nerve palsy, Movement disorder	Frontal lobe, Falx cerebri	Metastasis, Meningioma, Lymphoma	Antiepileptic	Stable disease
Krbanjevic 2021 [85]	50 F	Headache, Cognitive changes, Visual disturbance	Parietal lobe, Frontal lobe, Lateral ventricles	Meningioma, Metastasis, Hemangiopericytoma, Lymphoma, Sarcoidosis	Chemotherapy (nonspecific), Radiation therapy	Stable disease
Krishnamoorthy 2011 [86]	51 M	Fever, Ataxia/gait disturbance, Visual disturbance, Cranial nerve palsy	Frontal lobe	Meningioma	Corticosteroids	Partial recovery
Krueger 2019 [87]	52 F	Headache	Temporal lobe, Frontal lobe		Radiation therapy	Full recovery
Ksheerasagar 2020 [88]	38 F	Movement disorder, Visual disturbance, Cranial nerve palsy	Medulla, Pons, Cerebello-pontine angle	Lymphoma, Meningioma	Radiation therapy, Vinblastine, Chemotherapy (nonspecific), Corticosteroids	Stable disease
Kumar 2008 [89]	45 M	Headache, Seizure	Temporal lobe, Parietal lobe	Subdural hematoma, Meningioma		Full recovery
Kutty 2015 [91]	31 M	Seizure, Movement disorder	Frontal lobe, Parietal lobe, Dura	Meningioma, Langerhans cell histiocytosis, Metastasis		Partial recovery
LeGuenno 2012 [93]	57 M	Seizure	Frontal lobe, Temporal lobe		Corticosteroids, Antiepileptic	Stable disease
Leung 2003 [92]	35 M	Seizure	Parietal lobe		Corticosteroids	Full recovery
Li 2012 [96]	40 M	Headache, Cognitive changes	Occipital lobe			Full recovery
Li 2018 [97]	46 F	Numbness, Headache, Cranial nerve palsy	Cavernous Sinus	Meningioma		Partial recovery
Li 2021 [95]	26 F	Visual disturbance, Nausea/vomiting, Cranial nerve palsy, Polyuria/polydipsia, Weight gain	Suprasellar, Hypothalamus	Glioneural Tumor	Chemotherapy (nonspecific)	
Li 2022 [94]	23 F	Headache	Dura			
Lou 2012 [98]	27 M	Headache, Visual disturbance	Sellar, Suprasellar			
Lou 2012 [98]	29 M	Headache, Visual disturbance, Seizure	Sellar, Suprasellar			
Lou 2012 [98]	26 F	Headache, Visual disturbance, Nausea/vomiting	Sellar, Suprasellar			
Lou 2012 [98]	22 F	Headache, Visual disturbance	Sellar, Suprasellar			
Lu 2012 [99]	48 F	Painless mass	Frontal lobe	Meningioma		Full recovery
Mahzoni 2012 [100]	33 M	Headache, Ataxia/gait disturbance	Ventricular, Parietal lobe, Temporal lobe	Glioma, Lymphoma	Corticosteroids	Partial recovery
McPherson 2006 [103]	53 M	Headache, Visual disturbance, Movement disorder, Cranial nerve palsy	Sellar, Medulla, Cerebello-pontine angle, Pons	Meningioma	Corticosteroids	Stable disease
Mourad 2025 [104]	45 F	Seizure	Parietal lobe	Meningioma	Corticosteroids	Full recovery
Nóbrega 2021 [111]	38 M	Visual disturbance, Headache, Cranial nerve palsy	Cavernous Sinus, Frontal lobe, Temporal lobe, Cerebello-pontine angle		Corticosteroids	
Nakagawa 2020 [105]	39 M	Headache	Lateral ventricles		Corticosteroids	Progressive disease
Nalini 2012 [106]	35 M	Headache, Cranial nerve palsy, Visual disturbance	Suprasellar, Optic Nerves/Chiasm	Tuberculosis	Corticosteroids, Radiation therapy, Other	Progressive disease
Nasrollahi 2024 [107]	83 M	Headache	Superior Sagittal Sinus, Falx cerebri	Meningioma, Metastasis	Radiation therapy	Stable disease
Natarajan 2000 [108]	45 F	Seizure	Frontal lobe	Metastasis, Glioma		Full recovery
Navarro-Olvera 2023 [109]	59 M	Visual disturbance, Cranial nerve palsy, Seizure	Sellar	Meningioma	Corticosteroids	Full recovery
Ng 1995 [110]	22 M	Polyuria/polydipsia	Pituitary gland, Sellar	Other, Diabetes insipidus	Corticosteroids	Full recovery
Oo 2024 [112]	74 F	Numbness, Movement disorder, Cranial nerve palsy, Seizure	Parietal lobe, Frontal lobe, Dura, Hypothalamus, Temporal lobe	Meningioma	Corticosteroids, Antiepileptic, Chemotherapy (nonspecific)	Death
Ouazzani 2023 [113]	26 M	Nausea/vomiting, Dizziness/Vertigo, Headache, Visual disturbance, Cranial nerve palsy	Parietal lobe, Occipital lobe, Frontal lobe	Meningioma, Metastasis, Sarcoidosis	Corticosteroids	Full recovery
Parkhi 2024 [115]	59 M		Skull base		Radiation therapy	
Parkhi 2024 [115]	21 M		Dura, Foramen Magnum		Radiation therapy	
Parkhi 2024 [115]	31 M		Parietal lobe		Radiation therapy	
Parkhi 2024 [115]	55 M		Parietal lobe		Radiation therapy	
Pattnaik 2023 [116]	50 M	Headache, Seizure, Movement disorder	Parietal lobe	Meningioma		Full recovery
Patwardhan 2018 [117]	40 F	Headache	Temporal lobe, Lateral ventricles	Meningioma		Full recovery
Petraglia 2020 [118]	46 M	Headache, Nausea/vomiting, Movement disorder, Weight Loss	Pons, Medulla	Metastasis, Autoimmune disease, Sarcoidosis		Progressive disease
Petzold 2001 [119]	78 M	Ataxia/gait disturbance	Optic Nerves/Chiasm		Radiation therapy	Death
Petzold 2001 [119]	47 M	Visual disturbance, Headache	Foramen Magnum, Sphenoid, Cerebellopontine angle, Sellar	Meningioma	Radiation therapy	Stable disease
Prayson 2014 [120]	28 M	Seizure	Dura	Meningioma	Antiepileptic	Full recovery
Purav 2005 [121]	18 M	Weakness, Numbness	Skull base	Meningioma, Inflammation, Infection		Full recovery
Purav 2005 [121]	23 M	Nerve Palsy, Movement disorder	Middle fossa			Stable disease
Purav 2005 [121]	31 M	Seizure, Nerve Palsy	Parietal lobe			Stable disease
Purav 2005 [121]	37 M	Seizure	Parietal lobe, Occipital lobe			Stable disease
Purav 2005 [121]	37 M	Movement disorder, Seizure, Nerve Palsy	Parietal lobe			Stable disease
Purav 2005 [121]	39 M	Seizure	Frontal lobe			Stable disease
Purav 2005 [121]	50 M	Nerve Palsy, Movement disorder, Cranial nerve palsy	Parietal lobe			Death
Purav 2005 [121]	51 F	Headache, Seizure	Frontal lobe			Stable disease
Purav 2005 [121]	56 M	Seizure, Weakness	Parietal lobe			Stable disease
Purav 2005 [121]	60 F	Seizure	Parietal lobe			Stable disease
Qin 2023 [122]	43 M	Movement disorder	Cerebellum, Parietal lobe, Frontal lobe	Meningioma	Corticosteroids	Stable disease
Raslan 2008 [123]	50 M	Visual disturbance, Cranial nerve palsy	Frontal lobe, Temporal lobe	, Meningioma	Corticosteroids, Chemotherapy (nonspecific)	Progressive disease
Raslan 2011 [124]	50 M	Headache, Visual disturbance, Movement disorder	Temporal lobe			
Raslan 2011 [124]	54 M	Seizure, Visual disturbance, Cranial nerve palsy	Suprasellar, Sellar		Corticosteroids	
Raslan 2011 [124]	50 F	Headache, Other	Pituitary gland		Radiation therapy	
Ravi 2024 [125]	34 F	Visual disturbance, Headache, Cognitive changes, Cranial nerve palsy	Parietal lobe	Meningioma	Corticosteroids	Full recovery
Resnick 1996 [126]	38 M	Visual disturbance, Headache, Nausea/vomiting, Cranial nerve palsy	Cerebello-pontine angle		Corticosteroids	Stable disease
Reynolds 2015 [127]	46 F	Headache, Cognitive changes, Movement disorder	Insula	Meningioma, Metastasis, Hemangiopericytoma	6-mercaptopurine, Antiepileptic	Full recovery
Rezaei 2020 [128]	47 M	Dizziness/Vertigo, Seizure, Movement disorder	Parietal lobe, Dura	Meningioma, Metastasis	Corticosteroids, Antiepileptic	Full recovery
Riccio 2021 [129]	69 F	Headache, Nausea/vomiting, Dizziness/Vertigo, Visual disturbance, Cranial nerve palsy	Frontal lobe, Parietal lobe, Pons, Medulla, Cerebellum	Metastasis	Corticosteroids	Death
Richardson 2018 [130]	64 F	Visual disturbance, Cranial nerve palsy	Cerebellum, Cerebello-pontine angle, Temporal lobe	Multiple Sclerosis, Sarcoidosis	Corticosteroids	Death
Rivera 2014 [131]	22 M	Visual disturbance, Headache, Seizure	Parietal lobe		Chemotherapy (nonspecific), Other	Stable disease
Rivera 2014 [131]	39 M	Headache, Seizure	Frontal lobe, Temporal lobe		Chemotherapy (nonspecific)	Full recovery
Rotondo 2010 [132]	63 F	Ataxia/gait disturbance, Weight Loss	Dura, Pituitary gland, Pituitary stalk	Other, Intestinal perforation		Death
Russo 2009 [133]	71 M	Movement disorder, Nerve Palsy	Parietal lobe, Frontal lobe			Full recovery
Russo 2009 [133]	72 M	Headache	Frontal lobe	Lymphoma	Antiepileptic	Full recovery
Safi 2020 [134]	28 F	Headache, Weakness	Parietal lobe	Langerhans cell histiocytosis		Full recovery
Safi 2020 [134]	25 M	Loss of Consciousness	Frontal lobe	Lymphoma, Other		Full recovery
Safi 2020 [134]	25 M	Weakness	Other	Lymphoma, Infection, Granuloma		Stable disease
Safi 2020 [134]	52 M	Nerve Palsy, Movement disorder	Occipital lobe, Other	Lymphoma		Full recovery
Safi 2020 [134]	40 M	Nerve Palsy, Movement disorder	Other			Full recovery
Said 2011 [135]	74 M	Dizziness/Vertigo, Seizure, Movement disorder, Cranial nerve palsy, Cognitive changes	Parietal lobe, Temporal lobe	Meningioma, Subdural hematoma, Lymphoma	Corticosteroids	Death
Sakai 1998 [136]	60 M	Tinnitus, Cranial nerve palsy	Cerebellum, Pons, Frontal lobe, Temporal lobe	Meningioma		Full recovery
Sandoval-Sus 2014 [137]	32 F	Headache, Visual disturbance	Extra axial		Chemotherapy (nonspecific), Radiation therapy	Stable disease
Sandoval-Sus 2014 [137]	51 M	Cranial nerve palsy, Other	Extra axial		Radiation therapy	Stable disease
Sandoval-Sus 2014 [137]	53 M	Movement disorder, Ataxia/gait disturbance	Extra axial		Radiation therapy, Corticosteroids	Progressive disease
Sandoval-Sus 2014 [137]	18 M	Movement disorder, Spasticity	Extra axial		Chemotherapy (nonspecific), Vinblastine, Methotrexate, 6-mercaptopurine, Corticosteroids, Radiation therapy	Stable disease
Sandoval-Sus 2014 [137]	38 M	Other	Extra axial			Stable disease
Sandoval-Sus 2014 [137]	60 M	Visual disturbance, Cognitive changes	Parietal lobe, Occipital lobe			Full recovery
Sasidharan 2020 [138]	32 F	Weakness, Visual disturbance, Headache	Suprasellar, Medulla	Meningioma, Metastasis	6-mercaptopurine, Methotrexate, Radiation therapy	Full recovery
Sasidharan 2020 [138]	29 M	Visual disturbance	Sellar, Cavernous Sinus		Radiation therapy	Partial recovery
Sato 2023 [139]	68 F	Painless mass	Parietal lobe	Metastasis		Progressive disease
Seyednejad 2007 [140]	43 F	Movement disorder	Other, Parasellar, Dura		Corticosteroids	Progressive disease
Shah 2020 [141]	63 F	Headache, Visual disturbance, Cranial nerve palsy	Optic Nerves/Chiasm	Meningioma	Cladribine, Corticosteroids	Stable disease
Sharma 2005 [142]	40 M	Seizure	Frontal lobe, Temporal lobe	Meningioma	Antiepileptic	Full recovery
Shimizu 2024 [143]	64 M	Ataxia/gait disturbance, Other	Parietal lobe, Other, Occipital lobe	Meningioma, Other	Corticosteroids	Partial recovery
Shin 2022 [144]	65 M	Seizure, Movement disorder	Frontal lobe	Meningioma, Langerhans cell histiocytosis, Metastasis	Antiepileptic	Full recovery
ShuangshotiJr 1999 [145]	55 F	Visual disturbance	Frontal lobe, Temporal lobe	Meningioma	Corticosteroids	Partial recovery
Siadati 2001 [146]	48 F	Seizure	Parietal lobe, Occipital lobe, Dura	Meningioma		
Simos 1998 [147]	62 M	Seizure, Headache	Parietal lobe	Meningioma	Corticosteroids	Full recovery
Smith 2018 [148]	20 M	Headache, Cranial nerve palsy, Enlarged head, Painless mass	Parietal lobe	Lymphoma, Meningioma	Corticosteroids, Chemotherapy (nonspecific), Radiation therapy	Progressive disease
Song 1989 [149]	30 M	Seizure, Cranial nerve palsy, Cognitive changes	Optic Nerves/Chiasm, Pons, Cerebellum, Sellar, Parasellar, Cerebello-pontine angle, Medulla	Meningioma	Corticosteroids	Stable disease
Soto-Davila 2023 [150]	61 F	Dizziness, Headache, Other	Parietal lobe, Cerebellum	Meningioma		Full recovery
Swor 2021 [151]	31 F	Seizure	Suprasellar, Other, Temporal lobe, Falx cerebri	Eclampsia, Embolism, Sepsis	Antiepileptic	Stable disease
Symss 2010 [152]	21 M	Headache, Nausea/vomiting	Cavernous Sinus, Tenorital	Meningioma	Other, Radiation therapy	Progressive disease
Symss 2010 [152]	35 M	Seizure	Dura, Falx cerebri	Meningioma		Full recovery
Türe 2004 [163]	29 M	Visual disturbance, Other	Suprasellar, Other	Other, Langerhans cell histiocytosis	Corticosteroids, Chemotherapy (nonspecific), Other	Full recovery
Tan 2018 [153]	66 M	Cranial nerve palsy, Tinnitus	Temporal lobe	Meningioma, Lymphoma, Metastasis		Partial recovery
Tang 2020 [154]	67 M	Numbness, Movement disorder	Parietal lobe	Metastasis		Full recovery
Tatit 2021 [155]	37 M	Other, Tender lump, Movement disorder	Frontal lobe	Autoimmune disease	Corticosteroids	Full recovery
Tatit 2021 [155]	45 M	Headache, Visual disturbance, Cranial nerve palsy	Posterior fossa	Meningioma	Chemotherapy (nonspecific), Radiation therapy	Partial recovery
Taufiq 2016 [156]	24 M	Cranial nerve palsy	Optic Nerves/Chiasm, Parasellar	Meningioma	Corticosteroids	Partial recovery
Tauziede-Espariat 2015 [157]	35 F	Headache	Occipital lobe, Meninges	Other, Autoimmune disease	Corticosteroids	Full recovery
Tavangar 2006 [158]	79 M	Seizure, Headache, Fever	Dura, Parasellar, Leptomeningeal	Meningioma, Metastasis	Corticosteroids	
Tian 2015 [159]	26 M	Headache, Numbness	Middle fossa, Posterior fossa	Meningioma		Full recovery
Tian 2015 [159]	49 F	Seizure	Falx cerebri, Parietal lobe	Meningioma		Progressive disease
Tian 2015 [159]	68 M	Painless mass	Frontal lobe	Meningioma		Full recovery
Toh 2005 [160]	60 F	Dizziness/Vertigo	Cerebellum			Full recovery
Toh 2005 [160]	59 M	Seizure, Weakness	Frontal lobe			Stable disease
Tomio 2012 [161]	53 M	Seizure	Parietal lobe	Meningioma		Full recovery
Trudel 1984 [162]	28 M	Numbness, Headache, Ataxia/gait disturbance, Cranial nerve palsy	Dura	Meningioma	Radiation therapy	Partial recovery
Tyagi 2024 [164]	35 M		Meninges, Sphenoid	Tuberculosis	Corticosteroids	Progressive disease
Tyagi 2024 [164]	55 M		Sphenoid, Orbit	Other	Corticosteroids, Radiation therapy	Stable disease
Tyagi 2024 [164]	18 M		Cerebellum, Orbit	Other		Death
Tyagi 2024 [164]	49 M		Cerebello-pontine angle, Sphenoid	Other	Corticosteroids	Stable disease
Tyagi 2024 [164]	45 M		Frontal lobe	Meningioma		Full recovery
Tyagi 2024 [164]	33 M		Tenorital	Tuberculosis	Corticosteroids	Progressive disease
Tyagi 2024 [164]	25 F		Cerebellum, Falx cerebri	Meningioma	Radiation therapy	Stable disease
Tyagi 2024 [164]	28 M		Sphenoid, Orbit	Meningioma	Corticosteroids	Stable disease
Tyagi 2024 [164]	40 F		Cerebello-pontine angle, Middle fossa, Skull base	Meningioma	Steroid therapy	Stable disease
Tyagi 2024 [164]	29 F		Cerebellum, Medulla	Mutliple Sclerosis	Corticosteroids	Full recovery
Tyagi 2024 [164]	34 M		Middle fossa	Meningioma		Stable disease
Tyagi 2024 [164]	29 F		Tenorital	Meningioma	Corticosteroids	Full recovery
Tyagi 2024 [164]	27 M		Frontal lobe	Tuberculosis	Corticosteroids	Stable disease
Tyagi 2024 [164]	43 M		Sphenoid	Meningioma	Chemotherapy (nonspecific)	Full recovery
Tyagi 2024 [164]	19 F		Orbit, Occipital lobe, Sellar, Suprasellar	Glioma, Other	Corticosteroids	Progressive disease
Tyagi 2024 [164]	56 M		Frontal lobe	Glioma, Metastasis	Radiation therapy	Full recovery
Tyagi 2024 [164]	19 M		Temporal lobe, Insula	Lymphoma	Radiation therapy	Full recovery
Tyagi 2024 [164]	42 M		Parietal lobe	Other		Progressive disease
Tyagi 2024 [164]	34 M		Temporal lobe	Meningioma		Full recovery
Tyagi 2024 [164]	50 M		Skull base	Meningioma	Corticosteroids, Radiation therapy	Stable disease
Tyagi 2024 [164]	34 M		Parietal lobe, Occipital lobe	Meningioma	Radiation therapy	Stable disease
Udono 1999 [165]	67 F	Cranial nerve palsy	Frontal lobe, Dura	Meningioma		Full recovery
Unadkat 2022 [166]	55 M	Headache, Cranial nerve palsy	Cavernous Sinus, Suprasellar, Parasellar	Metastasis		Stable disease
Vaidya 2020 [167]	57 F	Weight Loss, Fever, Visual disturbance	Sellar, Suprasellar, Medulla			
Vaidya 2020 [167]	32 F	Visual disturbance, Headache	Sellar, Suprasellar, Parasellar			
Vaidya 2020 [167]	29 M	Visual disturbance	Sellar, Parasellar			
Vaidya 2020 [167]	45 M	Neck Swelling, Nasal Obstruction	Paranasal Sinus, Dura			
Vaidya 2020 [167]	19 M	Visual disturbance	Orbit, Paranasal Sinus			
Varrassi 2021 [168]	78 M	Movement disorder	Frontal lobe			
Varrassi 2021 [168]	57 M	Visual disturbance, Tinnitus, Cranial nerve palsy	Extra axial			Stable disease
Varrassi 2021 [168]	69 M	Cognitive changes	Parietal lobe, Temporal lobe, Occipital lobe	Glioma		Stable disease
Varrassi 2021 [168]	33 M	Headache, Nausea/vomiting, Ataxia/gait disturbance	Cerebellum			Full recovery
Wahba 2013 [169]	78 F	Headache, Dizziness/Vertigo, Ataxia/gait disturbance	Cerebellum	Meningioma, Lymphoma, Metastasis, Langerhans cell histiocytosis	Corticosteroids	Partial recovery
Walker 2011 [170]	54 F	Headache, Fever, Movement disorder	Dura	Lymphoma, Meningioma, Metastasis	Corticosteroids	Progressive disease
Wan 2008 [171]	43 M	Visual disturbance, Headache	Suprasellar	Meningioma		Full recovery
Wang 2010 [172]	22 M	Weakness	Parietal lobe	Meningioma		Full recovery
Wang 2010 [172]	40 F	Headache	Middle fossa	Meningioma		Full recovery
Wang 2010 [172]	38 M	Visual disturbance, Cranial nerve palsy	Orbit	Meningioma		Full recovery
Wang 2010 [172]	47 M	Cranial nerve palsy	Temporal lobe	Meningioma		Full recovery
Wang 2010 [172]	26 M	Seizure	Occipital lobe	Meningioma		Full recovery
Wang 2011 [176]	27 M	Polyuria/polydipsia	Sellar, Optic Nerves/Chiasm		Corticosteroids	Full recovery
Wang 2019 [173]	57 F	Visual disturbance, Cranial nerve palsy	Dura, Parasellar, Temporal lobe	Meningioma		Stable disease
Wang 2019 [177]	26 F	Headache, Nausea/vomiting	Dura, Pons, Medulla	Meningioma	, Other	Stable disease
Wang 2020 [174]	50 M	Visual disturbance, Cranial nerve palsy	Meninges		Corticosteroids, Chemotherapy (nonspecific)	Partial recovery
Wang 2020 [174]	29 M	Painless mass	Meninges		Corticosteroids, Chemotherapy (nonspecific)	Partial recovery
Wang 2020 [174]	37 M	Visual disturbance	Sellar			Stable disease
Wang 2023 [175]	19 M	Headache, Tinnitus, Ataxia/gait disturbance, Other, Cranial nerve palsy	Pons	Meningioma		Stable disease
Wang 2023 [175]	55 F	Headache, Visual disturbance	Parasellar	Lymphoma	Chemotherapy (nonspecific)	Full recovery
Wen 2019 [178]	54 F	Headache	Frontal lobe	Other		Full recovery
Wen 2019 [178]	40 M	Dizziness/Vertigo	Occipital lobe	Meningioma		Full recovery
Wen 2019 [178]	54 M	Seizure	Falx cerebri	Meningioma		Full recovery
Wrzolek 2002 [179]	38 M	Seizure	Frontal lobe	Meningioma		Full recovery
Wrzolek 2002 [179]	69 F	Headache	Cerebellum	Meningioma		Full recovery
Wu 2001 [180]	35 M	Headache, Seizure, Cognitive changes, Visual disturbance	Dura, Occipital lobe, Temporal lobe	Meningioma		Stable disease
Wu 2025 [181]	65 F	Visual disturbance	Frontal lobe, Sellar	Meningioma		Full recovery
Xia 2021 [182]	57 M	Dizziness/Vertigo, Visual disturbance	Temporal lobe	Meningioma		Full recovery
XiaoWen 2010 [183]	38 M	Visual disturbance	Dura, Meninges, Suprasellar, Sellar, Parasagittal, Temporal lobe	Meningioma, Sarcoidosis, Metastasis	Corticosteroids	Partial recovery
Xu 2024 [184]	28 F	Headache, Fever, Visual disturbance, Cognitive changes	Dura	Hypertrophic pachymeningitis	Corticosteroids	Stable disease
Yamauchi 2012 [185]	67 F	Other, Dizziness/Vertigo	Frontal lobe	Meningioma		Full recovery
Yazbeck 2023 [186]	24 M	Visual disturbance	Suprasellar, Cavernous Sinus, Temporal lobe	Meningioma	Corticosteroids, Radiation therapy	Full recovery
Z'Graggen 2006 [187]	35 M	Headache	Dura, Superior Sagittal Sinus, Falx cerebri	Meningioma	Corticosteroids	Full recovery
Zhang 2010 [188]	27 M	Headache, Visual disturbance, Cranial nerve palsy	Sellar	Meningioma		Full recovery
Zhang 2010 [188]	38 F	Headache, Nausea/vomiting	Pituitary gland, Suprasellar			Full recovery
Zhang 2010 [188]	26 F	Headache, Nausea/vomiting, Fever	Pituitary gland, Suprasellar	Tuberculosis		Full recovery
Zhang 2010 [188]	30 M	Headache, Nasal Obstruction, Epistaxis	Cavernous Sinus, Paranasal Sinus			Full recovery
Zhang 2022 [189]	67 M	Seizure	Frontal lobe, Temporal lobe	Meningioma		Partial recovery
Zhang 2022 [189]	49 M	Seizure	Frontal lobe, Other	Meningioma		Full recovery
Zhang 2022 [189]	46 M	Headache	Occipital lobe, Other	Meningioma		Full recovery
Zhang 2022 [189]	49 M	Numbness, Other	Parietal lobe, Other	Meningioma	Chemotherapy (nonspecific)	Full recovery
Zhang 2022 [189]	25 M	Headache, Other	Frontal lobe, Temporal lobe	Meningioma		Full recovery
Zhang 2022 [189]	49 M	Numbness, Other	Cerebellum, Other	Meningioma	Steroid therapy	Progressive disease
Zhang 2022 [189]	40 F	Headache, Dizziness	Sellar, Parasellar	Meningioma		Stable disease
Zhang 2022 [189]	51 M	Dizziness, Other	Parasellar, Other	Meningioma		Death
Zhang 2022 [189]	35 M	Other	Other	Meningioma		Stable disease
Zhang 2022 [190]	54 M	Seizure, Loss of Consciousness	Parietal lobe, Superior Sagittal Sinus	Meningioma	Valproate	Full recovery
Zheng 2023 [191]	58 M	Loss of Consciousness, Other, Seizure	Frontal lobe			Full recovery
Zhenwei 2025 [192]	48 F	Painless mass	Superior Sagittal Sinus	Meningioma, Erdheim-Chester disease, Langerhans cell histiocytosis, Lymphoma		Full recovery
Zhou 2021 [194]	47 M	Numbness	Frontal lobe, Parietal lobe, Temporal lobe	Meningioma, Metastasis		
Zhou 2021 [194]	19 M	Headache, Cranial nerve palsy	Cerebello-pontine angle	Meningioma, Metastasis	Radiation therapy	Partial recovery
Zhou 2022 [193]	32 M	Headache	Other	Subdural hematoma, Lymphoma, Metastasis		Full recovery
Zhu 2012 [196]	25 M	Headache, Seizure	Falx cerebri, Frontal lobe			
Zhu 2012 [196]	38 F	Headache, Dizziness/Vertigo, Visual disturbance	Dura, Temporal lobe			
Zhu 2012 [196]	46 M	Cognitive changes	Temporal lobe, Dura			
Zhu 2012 [196]	26 M	Seizure, Cognitive changes	Occipital lobe, Dura			
Zhu 2012 [196]	41 M	Headache, Seizure	Temporal lobe, Dura			
Zhu 2012 [196]	40 M	Visual disturbance, Nerve Palsy	Parasellar, Dura			
Zhu 2012 [196]	68 M	Headache	Frontal lobe, Superior Sagittal Sinus, Falx cerebri			
Zhu 2012 [196]	35 M	Headache, Visual disturbance	Occipital lobe			
Zhu 2012 [196]	47 M	Weakness	Parietal lobe, Falx cerebri, Dura			
Zhu 2013 [197]	54 M	Seizure, Dizziness/Vertigo	Parietal lobe, Occipital lobe		NSAID	Stable disease
Zhu 2013 [197]	60 M	Headache, Nausea/vomiting	Cerebellum		NSAID	Stable disease
Zhu 2013 [197]	26 F	Nausea/vomiting, Headache, Fever	Pituitary gland, Sellar, Suprasellar		NSAID	Stable disease
Zhu 2013 [197]	38 M	Nausea/vomiting, Headache, Fever	Pituitary gland, Sellar, Suprasellar		NSAID	Stable disease
Zhu 2013 [197]	27 M	Headache, Epistaxis	Paranasal Sinus		Corticosteroids, NSAID	Stable disease
Zhu 2013 [197]	53 M	Movement disorder	Other		NSAID	Stable disease
Zhu 2022 [195]	49 F	Movement disorder, Seizure	Parietal lobe, Parasagittal	Meningioma, Lymphoma		Stable disease
Zhu 2022 [195]	59 M	Movement disorder	Parietal lobe	Meningioma, Lymphoma		Stable disease
Zhu 2022 [195]	50 M	Movement disorder, Seizure	Parietal lobe	Meningioma, Lymphoma		Stable disease
Zhu 2022 [195]	29 M	Loss of Consciousness	Temporal lobe	Meningioma, Lymphoma		Stable disease
Zhu 2022 [195]	61 M	Headache, Numbness, Weakness	Skull base	Meningioma, Lymphoma		Stable disease
Zhu 2022 [195]	53 F	Headache	Occipital lobe	Meningioma, Lymphoma	Corticosteroids	Stable disease
Zhu 2022 [195]	31 M	Loss of Consciousness	Temporal lobe	Meningioma, Lymphoma		Stable disease
Zhu 2022 [195]	63 M	Weakness	Parietal lobe	Meningioma, Lymphoma		Stable disease
Zhu 2022 [195]	25 M	Seizure, Loss of Consciousness	Temporal lobe, Occipital lobe	Meningioma, Lymphoma		Stable disease
Zhu 2022 [195]	35 M	Visual disturbance, Cranial nerve palsy	Medulla	Meningioma, Lymphoma		Death
Zhu 2022 [195]	28 F	Numbness	Parasellar	Meningioma, Lymphoma		Stable disease
Zhu 2022 [195]	37 M	Movement disorder	Skull base	Meningioma, Lymphoma		Stable disease

**Fig. 6:**
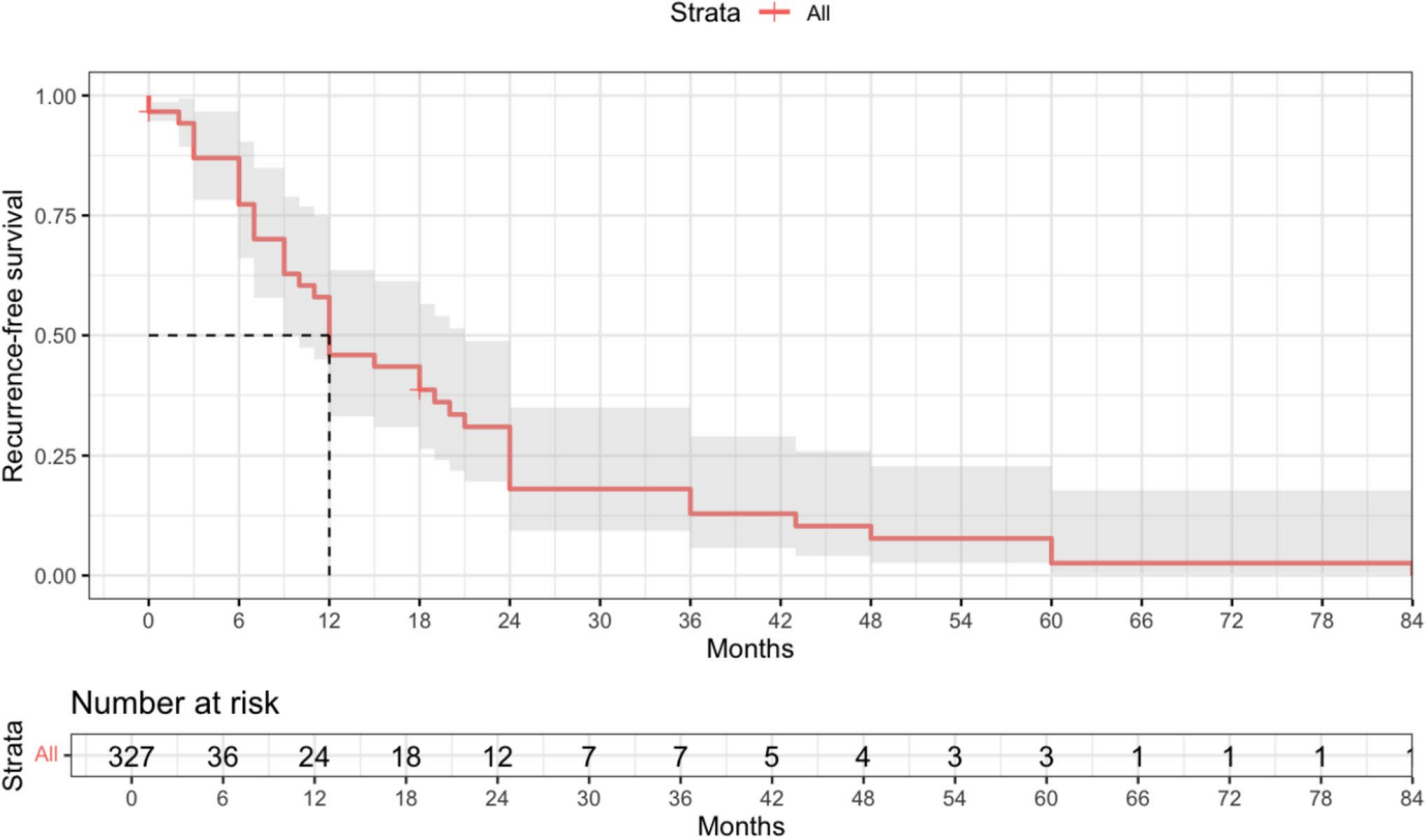
Survival probability curve for RFS (Recurrence Free Survival)

The recurrence-free survival (RFS) analysis demonstrated a median RFS of approximately 12 months, with RFS probability declining steadily thereafter (Fig. 6). By 24 months, fewer than one-third of patients remained recurrence-free, and by five years recurrence was observed in the vast majority of cases. These findings highlight the aggressive clinical course and underscore the importance of both initial disease control and long-term follow-up.

**Fig. 7:**
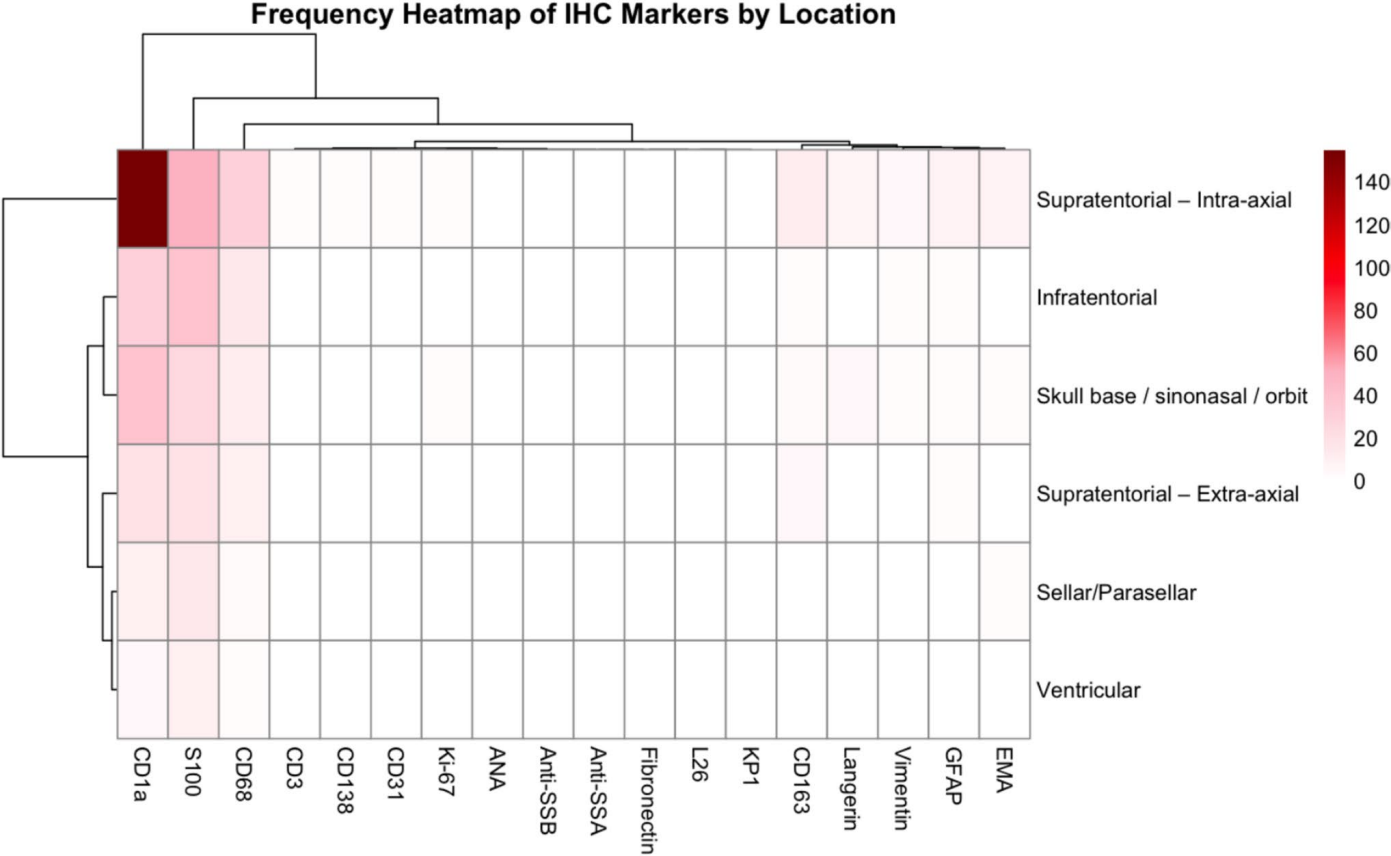
Distribution of IHC marker expression in relation to lesion location

The frequency heatmap of immunohistochemical (IHC) markers by lesion location showed that S100 and CD68 were the most frequently positive markers across nearly all anatomical sites, with CD1a positivity clustering more prominently in supratentorial intra-axial and skull base lesions (Fig. 7). In contrast, other markers such as GFAP, EMA, and CD163 were only sporadically expressed. This pattern supports the histopathological heterogeneity of the disease and highlights the diagnostic value of S100 and CD68 as consistent markers across locations.

## Discussion

This systematic review and IPD meta-analysis brings together the largest Intracranial Rosai–Dorfman disease (IC-RDD) cohort to date and situates these results alongside prior single center series and narrative reviews, clarifying points of consensus and uncertainty in the field. IC-RDD in our cohort predominantly affected men (70.9%) in middle adulthood (median 43.6 years) and most often presented with headache (35.2%), seizures or loss of consciousness (28.7%), and visual disturbance (26.9%). These findings mirror published IC-RDD series in which mass-effect symptoms dominate and dural-based lesions frequently masquerade as meningioma, while endocrine complaints are uncommon unless the sellar or parasellar region is involved. Headaches likely reflect dural nociceptor activation and venous sinus congestion, seizures arise from cortical irritation at the gray–white junction of supratentorial lesions, and visual symptoms map to compression/traction along the anterior visual pathway and optic radiations [60]. IC-RDD should also be considered within the wider spectrum of CNS histiocytoses, where diagnosis and treatment strategies are informed by disease chronicity, risk of recurrence, and evolving systemic therapies [38].

Topographically, disease clustered in the supratentorial compartment (intra-axial 52.9%, extra-axial 20.5%), with additional burdens in the sellar/parasellar (20.8%) and infratentorial (18.0%) regions. Our IPD showed location to be prognostically decisive: supratentorial intra-axial lesions were less likely to achieve full recovery (RR 0.56, 95% CI 0.41–0.75, *p* < 0.001), whereas extra-axial supratentorial (RR 1.87, 95% CI 1.17–2.99, *p* = 0.003), sellar/parasellar (RR 2.06, 95% CI 1.27–3.36, *p* < 0.001), and skull base or sinonasal or orbit disease (RR 1.88, 95% CI 1.04–3.42, *p* = 0.018) were associated with better recovery. Anatomically, extra-axial IC-RDD tends to respect arachnoid and dural planes and displace rather than infiltrate brain, enabling plane-based microsurgical resection; in contrast, intraparenchymal IC-RDD interdigitates with eloquent cortex and subcortical pathways, limiting safe margins and predisposing to residual disease and functional morbidity [41, 100, 152].

Imaging and tissue features were consistent with histiocytic pathology but underscored heterogeneity. MRI was the dominant modality (85%), and perilesional edema, present in 12.2%, predicted lower recovery (RR 0.65, 95% CI 0.47–0.89, *p* = 0.017), supporting the clinical relevance of the inflammatory microenvironment; hydrocephalus was rare (2.1%). Histologically, emperipolesis (57.5%) and sheets of S100-positive (87.5%), CD68-positive (64.5%) histiocytes anchored the diagnosis. Notably, CD1a positivity approached 47.4% in aggregated reports, higher than classic descriptions, likely reflecting site-specific immunophenotypic plasticity, small-biopsy sampling, or interpretive variability. CD1a positivity in presumed RDD can signal overlap with Langerhans cell histiocytosis. Correlating with Langerin and, when available, molecular testing helps reduce misclassification. From a neuropathological lens, correlating emperipolesis with S100 or CD68 co-expression and, when available, negative Langerin remains critical to distinguish IC-RDD from meningioma, inflammatory pseudotumor, and Langerhans-cell lesions in small intracranial samples [2, 3, 101, 102].

Management in this IC-RDD cohort was overwhelmingly surgical (93.6%), most commonly via craniotomy (76.1%), and both approach and extent of resection tracked outcomes. Craniotomy was associated with improved recovery (RR 0.44, 95% CI 0.27–0.71, *p* < 0.001), and GTR was strongly favorable (RR 0.26, 95% CI 0.19–0.37, *p *< 0.001), whereas subtotal/partial resections and biopsy-only strategies were linked to worse recovery. These observations align with neurosurgical principles for extra-axial histiocytic disease: removing the dural-based nidus and its inflamed capsule collapses the cytokine-rich interface responsible for edema and mass effect [10]. Adjuvant therapies (corticosteroids 25.1%, radiotherapy 13.5%, chemotherapy 6.7%) were used selectively for residual, unresectable, or multisite disease; their role within the skull base and cavernous sinus likely reflects constraints on radical resection where cranial nerve and ICA encasement limit surgical corridors [75].

Outcomes highlight meaningful relapse risk despite low mortality. Median RFS was around 12 months, with recurrence reported in 15–23% depending on numerator or denominator definitions and a median time to recurrence around 11–12 months; re-operation occurred in 7.0% and overall mortality was 4.9%. While historical narratives often label IC-RDD as “benign,” our data emphasize the need for vigilance, particularly after less-than-GTR or in intra-axial and cervicomedullary junction disease. Neuroanatomically, limited resection margins in eloquent cortex and along critical white-matter tracts, multifocal disease, as well as potential craniospinal axis seeding, plausibly drive earlier relapse and functional decline [45, 59]. This aligns with the observed RFS curve.

### Clinical implications for neurosurgeons

For adult IC-RDD, surgery remains the backbone of care, with maximal safe resection offering the best functional outcomes when anatomy permits. The natural history shows frequent relapse with RFS of about 12 months. Non-surgical therapy is used selectively for residual, unresectable, multifocal, or anatomically constrained disease, most often corticosteroids and radiotherapy, with occasional systemic agents. Looking ahead, management will likely emphasize risk-adapted strategies that pair maximal safe resection with selective adjuvant therapy and structured long-term surveillance.

### Study limitation

This review aggregates predominantly retrospective case reports or case series with heterogeneous reporting, introducing selection and publication bias and a geographic skew (notably China, India, and the USA). Outcome definitions, recurrence denominators, and follow-up were inconsistent, which may limit comparability and time-to-event inference. It is important to note that subgroup samples were small and underpowered, multiple comparisons increase false-positive risk, and duplicate patient reporting across publications cannot be fully excluded. There is also inherent bias toward surgically managed patients because cases without tissue confirmation are rarely published, and pathology interpretations were site-specific without centralized review, which may affect diagnostic consistency.

### Study novelty

Like previous literature, this work found that RDD has a slight male predominance, frequently presents mimicking a meningioma, and is often curable if treated correctly (127). This review expands on previous literature as it aggregates the latest findings on RDD from 327 cases across 33 different countries. Unlike prior work or smaller pooled series, our work is more generalizable, explores the clinical course of more patients post-op, and may thus better help support clinical decision making.

## Conclusion

Intracranial Rosai–Dorfman disease (IC-RDD) in this pooled cohort predominantly affected middle-aged men and presented with mass-effect symptoms; prognosis was strongly location-dependent, with supratentorial intra-axial disease portending poorer recovery while extra-axial, sellar or parasellar, and skull-base or orbital lesions fared better. Surgery was the mainstay of care, and GTR was the key modifiable factor associated with improved recovery, whereas perilesional edema signaled worse outcomes and recurrence was nontrivial. Histology remained anchored by emperipolesis with S100 or CD68 co-expression, underscoring the importance of careful molecular profiling to neuropathologically distinguish RDDfrom mimics. This work illustrates the value of multicenter registries in the characterization and treatment of RDD, increasing our understanding of the disease and informing best practices going forward. Taken together, these data support a management paradigm prioritizing maximal safe resection for extra-axial disease, selective adjuvant therapy for residual or unresectable or multisite involvement, and vigilant, long-term MRI surveillance to detect recurrence and inform future treatment.

## Supplementary Information

Below is the link to the electronic supplementary material.

ESM 1 Supplementary Material 1 (DOCX 7.47 KB)

## Data Availability

No datasets were generated or analysed during the current study.
