# Supplementary material for: Intracranial manifestations of adult Rosai-Dorfman disease: a systematic review and IPD meta-analysis of 327 cases
Source: Acta Neurochir (Wien). 2025 Dec 6;167(1):316. doi: 10.1007/s00701-025-06735-w (PMC12682919; doi:10.1007/s00701-025-06735-w)
Supplement: Supplementary file 1 — Supplementary Material 1 (DOCX 7.47 KB) [file 701_2025_6735_MOESM1_ESM.docx]

####

| **Database** | **Search String** | **Results** | **Date Searched** | **Filters** |
| --- | --- | --- | --- | --- |
| **PubMed** | ("Histiocytosis, Sinus"[Mesh] OR "Rosai-Dorfman*"[tiab] OR "RDD"[tiab] OR "Sinus histiocytosis with massive lymphadenopathy"[tiab]) AND ("Central Nervous System"[Mesh] OR "central nervous system"[tiab] OR "brain"[tiab] OR "intracranial"[tiab] OR "spin*"[tiab] OR "dura*"[tiab] OR "Neurosurgery"[Mesh] OR "neurosurgery"[tiab] OR "craniotomy"[tiab] OR "laminectomy"[tiab]) | 551 | 5/16/2025 | None |
| **Scopus** | TITLE-ABS-KEY("Rosai-Dorfman disease" OR "RDD" OR "sinus histiocytosis with massive lymphadenopathy") AND TITLE-ABS-KEY("central nervous system" OR "brain" OR "intracranial" OR "spin*" OR "dura*" OR "neurosurgery" OR "craniotomy" OR "laminectomy") | 785 | 5/16/2025 | None |
| **Cochrane** | ("Rosai-Dorfman disease" OR "RDD" OR "sinus histiocytosis with massive lymphadenopathy") AND ("central nervous system" OR "brain" OR "intracranial" OR "spinal" OR "dural" OR "neurosurgery" OR "craniotomy" OR "laminectomy") | 3 | 5/16/2025 | None |
